# Improving Gallbladder Cancer Outcomes with Antibody-Based Therapies and Immunological Profiling: A Literature Review

**DOI:** 10.3390/antib15030049

**Published:** 2026-06-16

**Authors:** Christian Caglevic, Mario Alex Contreras-Torrez, Felipe Reyes-Cosmelli, Rodrigo Uribe-Maturana, Mauricio Mahave, Nicole Caire, Luis Villanueva-Olivares, Fernando Cid, Alvaro Lladser, Jorge Sapunar

**Affiliations:** 1 Fundación Arturo López Pérez OECI Cancer Center—Centre of Research and Innovation in Cancer (CIIC-FALP), Santiago 7500966, Chile; felipe.reyes@falp.org (F.R.-C.); rodrigo.uribe@falp.org (R.U.-M.); mahavem@falp.org (M.M.); nicole.caire@falp.org (N.C.); luis.villanueva@falp.org (L.V.-O.); fernando.cid@falp.org (F.C.); jorge.sapunar@falp.org (J.S.); 2 Postgraduate Medicine Faculty, Universidad de Los Andes, Santiago 7500966, Chile; 3 Medical Oncology Fellow, Universidad de los Andes, Santiago 7500966, Chile; 4 Medical Oncology Unit, Hospital Clínico Universidad de Chile, Santiago 7500966, Chile; 5 Medical Oncology Unit, Hospital del Salvador, Santiago 7500966, Chile; 6 Centro Basal Ciencia & Vida, Fundación Ciencia & Vida, Santiago 7500966, Chile; alladser@cienciavida.org; 7 Faculty of Medicine, Universidad San Sebastián, Santiago 7500966, Chile; 8 Faculty of Medicine, Universidad de la Frontera, Temuco 4780000, Chile

**Keywords:** gallbladder cancer, biliary tract cancer, bispecific antibodies, antibody-drug conjugates, tumor microenvironment, diagnostic and therapeutic biomarkers, population genomics

## Abstract

Gallbladder cancer (GBC) is an aggressive tumor that, together with the cholangiocarcinomas, constitutes the spectrum of biliary tract cancer (BTC). These tumors are characterized by a frequently late diagnosis, marked genomic heterogeneity, variable response to cytotoxic therapies, and poor overall survival in advanced stages. Nevertheless, the characterization of the tumor microenvironment (TME) and the identification of actionable molecular targets have driven the development of biological therapies. This review summarizes current and emerging evidence on monoclonal antibodies, bispecific antibodies, and antibody–drug conjugates (ADCs) in the management of GBC. The analysis addresses the early exploration of autoantibodies as potential diagnostic biomarkers, mechanistic hypotheses of immune evasion, and the clinical translation of targeted agents in the metastatic setting. Additionally, we critically discuss the extrapolation of data from global BTC trials to the specific GBC setting, the integration of population genetics into epidemiological studies such as the EULAT Eradicate GBC initiative, and the preliminary status of immunotherapy in perioperative scenarios.

## 1. Introduction

Gallbladder cancer (GBC) is the most frequent malignancy of the biliary tract (BTC) [1]. In contrast to other geographic regions where it is considered a rare tumor, GBC shows a significant incidence in specific populations, particularly in Latin America and Asia [2]. Owing to the absence of submucosa in the gallbladder wall and its non-specific early symptomatology, a large proportion of patients present with metastatic or locally advanced unresectable disease [1]. In these stages, curative surgical resection is not feasible [3].

Over the last decade, first-line systemic treatment for locally advanced unresectable and metastatic disease has been based on chemotherapy regimens, specifically the cisplatin–gemcitabine doublet (Cis-Gem) [4]. Attempts to intensify conventional chemotherapy have not consistently translated into substantial clinical benefit. For instance, the randomized phase III SWOG S1815 trial (n = 441 eligible; ~31% with GBC) demonstrated that the addition of the taxane nab-paclitaxel to the cisplatin–gemcitabine regimen did not significantly improve overall survival (OS) in patients with newly diagnosed BTC: median OS of 14.0 months for the triplet (gemcitabine 800 mg/m^2^ + cisplatin 25 mg/m^2^ + nab-paclitaxel 100 mg/m^2^ IV; days 1 and 8 of 21-day cycles) versus 13.6 months for the standard doublet (gemcitabine 1000 mg/m^2^ + cisplatin 25 mg/m^2^ IV; days 1 and 8 of 21-day cycles); HR 0.91 (95% CI: 0.72–1.14; *p* = 0.41) [5]. Treatment was administered until disease progression or unacceptable toxicity (Table 1) [5,6]. In this context of marginal chemotherapeutic benefit, research has recently shifted toward molecular profiling and immunotherapy.

Currently, it is recognized that GBC harbors subgroups with actionable genetic aberrations and differentiated immunological phenotypes [6]. The development of targeted therapies, including antibodies against human epidermal growth factor receptor 2 (HER2/ERBB2), has opened new lines of investigation [7]. Moreover, the therapeutic landscape has expanded with the evaluation of bispecific molecules (targeting pathways such as PD-1 and TIGIT) [8] and antibody–drug conjugates (ADCs) [9].

This review synthesizes the clinical and translational evidence on antibody-based therapies in GBC. It is important to note that a large part of the therapeutic literature groups GBC within the global BTC category (together with intrahepatic and extrahepatic cholangiocarcinomas) [1]. This review is limited to results published in the GBC setting.

**Table 1:** Pivotal clinical trials in biliary tract cancer (BTC) and gallbladder cancer (GBC) discussed in this review.

Trial/NCT	Phase/Setting	Population (n; % GBC)	Experimental Arm	Comparator	Primary Endpoint	Key Efficacy Outcomes	Reference
ABC-02/	Phase III, 1L advanced/metastatic BTC	410 (149 GBC; 36%)	Cisplatin 25 mg/m^2^ + gemcitabine 1000 mg/m^2^ IV D1, D8 Q21d, up to 8 cycles	Gemcitabine 1000 mg/m^2^ IV D1, D8, D15 Q28d, up to 6 cycles	OS	mOS 11.7 vs. 8.1 mo (HR 0.64; 95% CI 0.52–0.80; *p* < 0.001); mPFS 8.0 vs. 5.0 mo; ORR 26%	[4]
BT22	Phase II randomized, 1L advanced BTC (Japan)	84 randomized, 83 treated (32 GBC; 39%)	Cisplatin 25 mg/m^2^ + gemcitabine 1000 mg/m^2^ IV D1, D8 Q21d (median 6 cycles)	Gemcitabine 1000 mg/m^2^ IV D1, D8, D15 Q28d (median 4 cycles)	OS	mOS 11.2 vs. 7.7 mo; mPFS 5.8 vs. 3.7 mo; HR for OS 0.69 (95% CI 0.42–1.13); ORR 19.5% vs. 11.9%	[10]
SWOG S1815/	Phase III, 1L newly diagnosed advanced BTC	441 (~31% GBC)	Gemcitabine 800 mg/m^2^ + cisplatin 25 mg/m^2^ + nab-paclitaxel 100 mg/m^2^ IV D1, D8 Q21d until PD/toxicity	Gemcitabine 1000 mg/m^2^ + cisplatin 25 mg/m^2^ IV D1, D8 Q21d until PD/toxicity	OS	mOS 14.0 vs. 13.6 mo (HR 0.91; 95% CI 0.72–1.14; *p* = 0.41); primary endpoint not met	[5]
TOPAZ-1/	Phase III randomized, double-blind, placebo-controlled, 1L advanced BTC	685 (~25% GBC)	Cisplatin 25 mg/m^2^ + gemcitabine 1000 mg/m^2^ IV D1, D8 Q21d (≤8 cycles) + durvalumab 1500 mg IV Q3W during chemo, then 1500 mg IV Q4W maintenance until PD	Same chemo + placebo	OS	mOS 12.8 vs. 11.5 mo (HR 0.80; 95% CI 0.66–0.97; *p* = 0.021); mPFS 7.2 vs. 5.7 mo	[11]
KEYNOTE-966/	Phase III randomized, double-blind, 1L advanced BTC	1069 (stratified by site: GBC vs. iCCA vs. eCCA; ~22% GBC)	Pembrolizumab 200 mg IV Q3W (≤35 cycles) + gemcitabine 1000 mg/m^2^ IV D1, D8 Q21d (until PD) + cisplatin 25 mg/m^2^ IV D1, D8 Q21d (≤8 cycles)	Same chemo + placebo	OS	mOS 12.7 vs. 10.9 mo (HR 0.83; 95% CI 0.72–0.95; one-sided *p* = 0.0034); mPFS 6.5 vs. 5.6 mo	[12]
KEYNOTE-158 + KEYNOTE-028 (pooled BTC cohort)/	Phase II non-randomized, pretreated advanced BTC, PD-L1+ (≥1% CPS)	128 BTC pooled (% GBC NR)	Pembrolizumab 200 mg IV Q3W (KN-158) or 10 mg/kg IV Q2W (KN-028) until PD or 24 mo	None	ORR	ORR 5.8–13%; mOS 7.4 mo (KN-158)/5.7 mo (KN-028); responses durable	[13]
SWOG S1609 DART (cohort 48)/	Phase II basket, refractory advanced GBC	19 (100% GBC)	Nivolumab 240 mg IV Q2W + ipilimumab 1 mg/kg IV Q6W until PD/toxicity	None	ORR	ORR 16% (3/19; 95% CI 5–34); 6-mo PFS 26%; mOS 7.0 mo (95% CI 3.9–19.1)	[14]
INTR@PID BTC 047/	Phase II single-arm, pretreated advanced BTC	159 (32 GBC; 20.1%)	Bintrafusp alfa 1200 mg IV Q2W until PD/toxicity	None	ORR	ORR 10.7% overall (95% CI 6.5–16.3); mOS 7.6 mo; mPFS 1.8 mo; primary endpoint not met	[15]
HERIZON-BTC-01 (cohort 1)	Phase IIb single-arm, pretreated HER2+ advanced BTC	87 enrolled; 80 in HER2+ cohort 1 (41 GBC; 51%)	Zanidatamab 20 mg/kg IV D1, D15 Q28d until PD/toxicity (median 6 cycles; median treatment duration 5.1 mo)	None	Confirmed ORR	ORR 41.3% (95% CI 30.4–52.8); ORR 51.6% in HER2 IHC 3+ vs. 5.6% in IHC 2+; DoR 12.9 mo; mPFS 5.5 mo	[16]
DESTINY-PanTumor02 (BTC cohort)/	Phase II open-label multicohort, pretreated HER2-expressing solid tumors	267 enrolled in Part 1; BTC cohort n = 41 (% GBC NR)	T-DXd 5.4 mg/kg IV Q3W until PD/toxicity (median 8.0 cycles; median duration 5.6 mo; 21.7% received ≥18 cycles)	None	ORR	ORR 22.0% in BTC overall; 56.3% in central HER2 IHC 3+ (9/16) vs. 18.8% IHC 2+; mPFS 4.6 mo (7.4 mo IHC 3+); mOS 7.0 mo (12.4 mo IHC 3+)	[17]
IMbrave 151/	Phase II randomized, double-blind, proof-of-concept, 1L advanced BTC	162 randomized (79 atezo+bev+CisGem; 83 atezo+placebo+CisGem); stratified by anatomical site (iCCA vs. eCCA vs. GBC)	Atezolizumab 1200 mg + bevacizumab 15 mg/kg + cisplatin 25 mg/m^2^ + gemcitabine 1000 mg/m^2^ IV Q3W	Atezolizumab + placebo + CisGem	PFS	mPFS 8.3 vs. 7.9 mo (HR 0.67; 95% CI 0.46–0.95); mOS 14.9 vs. 14.6 mo;	[18]
GAIN/	Phase III randomized open-label, perioperative resectable GBC/BTC	Planned 327 (target enrollment)	Neoadjuvant + adjuvant gemcitabine 1000 mg/m^2^ + cisplatin 25 mg/m^2^ IV D1, D8 Q21d (3 cycles pre-op + 3 cycles post-op) + surgery	Surgery + adjuvant chemo	OS	Ongoing; final results presented JCO 2025 abstr 4008	[19]
POLCAGB/	Phase III randomized open-label, locally advanced unresectable GBC	Planned ~138 (recruiting)	Neoadjuvant chemoradiation: gemcitabine + cisplatin + concurrent radiotherapy 45–55 Gy → surgery → adjuvant chemo	Neoadjuvant chemotherapy alone (gemcitabine + cisplatin) → surgery → adjuvant chemo	OS	Interim report shows resectability and pCR feasibility; final pending	[20]
BILCAP/	Phase III randomized open-label, adjuvant resected BTC	447 (84 GBC; 18.8%)	Capecitabine 1250 mg/m^2^ PO BID D1–14 Q21d × 8 cycles	Observation	OS	mOS 51.1 vs. 36.4 mo (per-protocol HR 0.75; 95% CI 0.58–0.97; *p* = 0.028); ITT non-significant	[21]

Abbreviations: BTC: biliary tree cancer; CPS: combined positive score; d: day; DoR: duration of response; eCCA: extrahepatic cholangiocarcinoma; GBC: gallbladder cancer; HR: hazard ratio; mo: months; iCCA: intrahepatic cholangiocarcinoma; ITT: intention to treat; NR: not reported L: line; mOS: median overall survival; mPFS: median progression free survival; ORR: overall response rate; OS: overall survival; pCR: pathological complete response; PD: progression disease; Q: interval in days.

## 2. Epidemiology, Ancestry, and Research Consortia

The epidemiology of GBC shows significant regional and ethnic disparities [22]. These differences suggest the complex interaction between genetic determinants and environmental or metabolic risk factors, such as chronic cholelithiasis [1].

### 2.1. Geographic Distribution

According to GLOBOCAN 2022, certain regions in the Andes and South Asia concentrate the highest disease burden [2,23]. Considering age-standardized incidence rates (ASR, per 100,000 inhabitants), countries such as Bolivia (overall ASR of 7.6) and Chile (ASR of 5.7) report the highest incidences worldwide [1,23]. When stratified by sex, women in these nations face a markedly higher risk (ASR of 8.4 in Bolivia and 7.4 in Chile). In contrast, the incidence in the United States and Europe typically ranges from 1.0 to 1.6 per 100,000 inhabitants, evidencing a substantial epidemiological gap [1,23].

### 2.2. Genetic Ancestry and Exploratory Molecular Characterization

Observational epidemiological studies have documented associations between genetic ancestry and GBC risk [24]. In South American cohorts, a correlation has been described between increasing proportions of Amerindian lineage and higher incidence. One study determined a 0.8% increase in GBC risk per 1% increment in individual Mapuche ancestry proportion in Chile [25]. In Bolivia, Aymara individuals presented an odds ratio (ORs) of 15.9 for GBC development compared with the mestizo population [26].

From a molecular perspective, genomic studies of GBC show variable frequencies. In retrospective studies of Chilean cohorts, *TP53* mutations have been reported in a range of 30% to 50% of samples [27]. Regarding predictive biomarkers, high microsatellite instability (MSI-H) is globally present in approximately 1% to 3% of BTC [1]. Although historical reports from limited cohorts in endemic areas of Chile described MSI-H frequencies of up to 10% in GBC, this figure reflects extreme regional variability and is not strictly generalizable [28].

Conversely the frequency of *ERBB2* alterations shows a comparable prevalence across geographically distinct cohorts, positioning it as a cross-population potential therapeutic target in GBC, as described by Mondaca et al. (9% in the Chilean cohort vs. 14% in the U.S. cohort; *p* = 0.42) [7]. Although previous series report ranges from 2% to 31%, contemporary cohorts profiled with validated NGS converge around 9–15%, which supports—with the caution imposed by methodological heterogeneity and sample-size imbalance—the relative consistency of this biomarker across populations. This positions HER2 as the most frequent therapeutic target for antibody development in this disease [7].

### 2.3. The EULAT Eradicate GBC Initiative

In response to the preventive gaps in high-incidence regions, the EULAT Eradicate GBC consortium has been established as a bi-continental initiative (Europe–Latin America) aimed at refining GBC prevention strategies. Through the projected recruitment of more than 14,000 participants and the consolidation of a centralized biorepository, the consortium seeks to identify epidemiological and molecular differences [29]. One of its main goals is the creation of multifactorial risk scores [24]. Preliminarily, internal models of the consortium suggest that the current number of 115 prophylactic cholecystectomies required in Chile to prevent one single GBC case could be reduced to 92 by incorporating information such as body mass index, educational level, and surnames of indigenous ancestry [30]. Although these predictive models are in the validation phase, they illustrate the effort required to ground precision prevention in high-risk populations. In this context, the high-risk population for GBC is operationally defined, according to Boekstegers et al., as individuals with gallstones at age 40 and an estimated risk above the median when integrating non-genetic factors (BMI > 25 kg/m^2^, primary-only education, Mapuche surnames, parity ≥ 3 in women, and family history of GBC) and, optionally, genetic factors (Mapuche ancestry proportion and rs17209837 genotype). This stratification reduces the number of cholecystectomies needed (NCNs) to prevent one GBC case by age 70 from 115 (95% CI: 104–131) in the baseline model—gallstones, sex, and year of birth—to 92 (95% CI: 60–128) with non-genetic factors and 80 (95% CI: 59–110) when genetic factors are added. The main risk determinants are gallstones (HR 5.74), parity ≥ 3 (HR 2.50), rs17209837 genotype (HR 1.42), primary education (HR 1.41), family history (HR 1.39), BMI (HR 1.03 per kg/m^2^), and Mapuche ancestry (HR 1.04 per 1%) [30].

## 3. Diagnostic Biomarkers and the Exploration of Autoantibodies

Early detection of GBC is an infrequent clinical scenario due to the asymptomatic presentation of the disease [1]. Conventional serum biomarkers, primarily CA 19-9 and CEA, present recognized limitations during screening. A recent prospective analysis of 1500 newly diagnosed patients with GBC showed that, although CA 19-9 and CEA were significantly higher in metastatic disease at presentation, their diagnostic performance for metastatic triage was limited by low sensitivity (40.3%), despite high specificity (89.1%) and a moderate area under the curve (AUC) of 0.74 [31].

In response to these limitations, basic research has explored endogenous autoantibodies as early sensors of malignancy. The appearance of neoantigens derived from mutated proteins can trigger peripheral humoral responses [32].

Current evidence on autoantibody signatures in GBC is strictly exploratory and hypothesis-generating. Initial studies have evaluated autoantibodies against intracellular proteins such as Annexin A1 (*ANXA1*). Although elevated serum levels were reported in patients with early GBC compared with lithiasis controls, the isolated sensitivity observed was only 41.7%, with a specificity of 89.9% and an AUC of 0.69. These metrics indicate that a single autoantibody lacks the operative performance required for clinical screening [32].

In parallel, plasma proteomics combined with regularized machine learning approaches have proposed discriminative multi-protein panels. In an exploratory retrospective study from Karolinska University Hospital (44 histologically confirmed GBC vs. 38 cholecystitis), preoperative plasma was profiled with the SomaScan^®^ 7K aptamer-based platform (7596 proteins screened) and processed with Elastic-Net (EN) and LASSO regularization, in an 80/20 training-test split with leave-one-out cross-validation. The EN model, applied to a 577-protein diagnosis-associated subset, achieved an AUC of 94% in the test set, whereas a more parsimonious 13-protein LASSO signature (PAHX, CD8A, HRG, CRIS2, AT2A3, ARY1, HMX2, VEGFsR2, DKK2, ITIH1, DEPP, dynactin sub2 and CSTN2) reached an AUC of 98%. The authors did not report sensitivity or specificity at predefined cut-offs and used histologically confirmed cholecystitis—not healthy controls—as a comparator, which represents the clinically relevant differential diagnosis in endemic regions, where cholecystitis is a high pretest-probability condition. The authors themselves acknowledge the absence of external validation and explicitly frame the results as exploratory. These findings are hypothesis-generating and represent an early-stage proof of concept that contrasts with the substantially lower performance of single-autoantibody markers such as ANXA1 (sensitivity 41.7%, specificity 89.9%, and AUC 0.69) and require prospective multi-institutional validation before any screening application [33].

## 4. The Tumor Microenvironment: Classifications and Mechanistic Hypotheses

**Figure 1:**
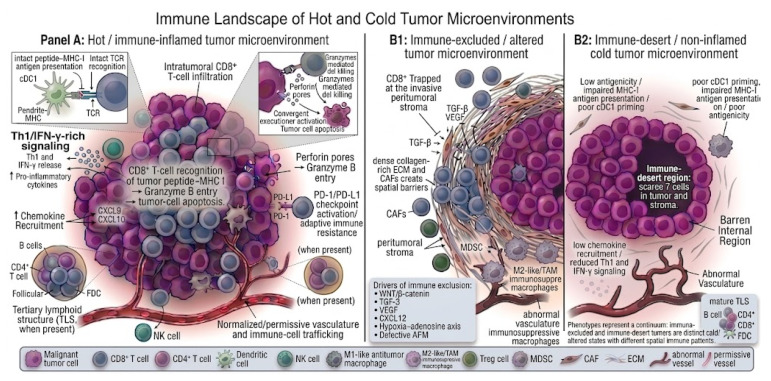
**Immune landscape of hot and cold tumor microenvironments.** (**A**): hot/immune-inflamed microenvironment with intact peptide–MHC class I antigen presentation by conventional type 1 dendritic cells (cDC1s), native TCR recognition, intratumoral CD8^+^ T-cell infiltration, Th1/IFN-γ-rich signaling, CXCL9/CXCL10-dependent chemokine recruitment, perforin- and granzyme B-mediated cytolytic execution, PD-1/PD-L1 checkpoint engagement as adaptive immune resistance and, when present, tertiary lymphoid structures. (**B1**) immune-excluded/altered microenvironment with CD8^+^ T cells trapped at the invasive peritumoral stroma, dense collagen-rich extracellular matrix, cancer-associated fibroblasts (CAFs), TGF-β/VEGF signaling, myeloid-derived suppressor cells (MDSCs) and M2-like/tumor-associated immunosuppressive macrophages; major drivers of exclusion include WNT/β-catenin, TGF-β, VEGF, CXCL12, the hypoxia–adenosine axis and defective AFM. (**B2**) immune-desert/non-inflamed cold microenvironment with low antigenicity, impaired MHC-I antigen presentation, poor cDC1 priming, abnormal vasculature and low chemokine recruitment with reduced Th1/IFN-γ signaling. These phenotypes represent a continuum and may coexist as distinct spatial states within different regions of an individual patient’s tumor.

The clinical efficacy of therapeutic antibodies in the biliary tract is modulated by the biology of its tumor microenvironment (TME). Transcriptomic studies propose the conceptual subdivision of BTC into opposing architectures, such as “stroma-rich” phenotypes and those rich in immune infiltrate [34]. The stroma-rich phenotype exhibits an intense desmoplastic reaction mediated by cancer-associated fibroblasts (CAFs) [34,35]. In vitro investigations suggest that this rigid matrix could hinder the infiltration of effector T cells and limit tissue penetration of systemic therapeutic macromolecules [35,36]. Additionally, translational studies have outlined potential mechanisms of immune evasion in the biliary tumor microenvironment [36,37]. In GBC, the reported prevalence of PD-L1 expression is highly variable, ranging from 9% to 72% depending on the methodology used. In the largest cohort published to date, Mody et al. analyzed 203 GBC cases by immunohistochemistry with the SP142 clone (Ventana), considering positivity as a score ≥ 2+ with ≥5% of stained tumor cells, and reported a positivity rate of 12% (25/203) [37]. It is important to emphasize that this criterion corresponds to a tumor proportion score (TC) and not to a combined positive score (CPS), which is the system used to define eligibility for immunotherapy in gastric or esophageal cancer. In the same cohort, PD-1 expression in tumor-infiltrating lymphocytes (TILs), evaluated with the NAT105 clone and a ≥1+ threshold, was substantially higher (55% in GBC), suggesting an immune microenvironment with adaptive but exhausted activation. The heterogeneity of clones, scoring systems, and thresholds employed across studies limits direct comparability of results and, consequently, the predictive value of PD-L1 for immunotherapy response in GBC remains incompletely defined (Table 2) [37]. The clinical relevance of this gap becomes evident when analyzing the TOPAZ-1 trial, which demonstrated the benefit of adding durvalumab to gemcitabine–cisplatin without requiring PD-L1-based selection, as randomization was stratified solely by disease stage and primary tumor location. This design contrasts with that of registration trials in adjacent tumors, in which specific CPS cut-offs are used to define eligibility. Accordingly, the development of robust predictive biomarkers in GBC will require harmonizing the methodology used to evaluate PD-L1 in translational studies with the way it is applied—or omitted—as a selection criterion in registration trials [38]. Other proposed mechanisms include cytokine secretion and metabolic reprogramming, in which stromal elements release mediators such as TGF-β or metabolites such as adenosine derived from eATP hydrolysis via CD39/CD73 [36]. In preclinical models, these pathways favor polarization toward immunosuppressive M2 macrophages and regulatory T cells (Tregs), functionally blocking the immune synapse [36]. It is relevant to emphasize that these hypotheses derive mainly from basic and translational studies [34,35,36] (Figure 1).

### Neoantigens, Peptide–MHC Presentation, and TCR-Mimic Antibody Strategies in Gallbladder Cancer

Neoantigens are tumor-specific peptides generated by somatic non-synonymous mutations, frameshift indels, gene fusions, or aberrant splicing events that, after intracellular processing, are presented by MHC class I and recognized as non-self by CD8^+^ T cells [39]. Their conceptual appeal—distinguishing them from tumor-associated antigens such as HER2—lies in their tumor-restricted expression and, consequently, a more favorable theoretical safety profile for T-cell-redirecting therapies, including TCR-engineered cells, neoantigen-directed vaccines, pMHC-directed bispecifics, and TCR-mimic ADCs [39,40].

The evidence base in GBC specifically remains immature. Comprehensive genomic characterization has shown that GBC harbors recurrent alterations in *TP53*, *ERBB2*/*ERBB3*, *SMAD4*, *ARID1A*, *KRAS*, *CDKN2A*, and members of the PI3K pathway [6,27,41], a mutational repertoire that is, in principle, capable of generating MHC-presentable neoepitopes. However, GBC tumor mutational burden is generally low to intermediate, and high microsatellite instability—one of the most robust surrogates of a neoantigen-rich phenotype—is reported in only ~1–3% of BTC overall, with regional series in endemic Chilean cohorts describing variable, non-generalizable frequencies. Direct functional evidence of neoantigen-specific T-cell recognition in GBC remains very limited; most available data are inferred from in silico HLA-binding predictions rather than from validated ELISpot, tetramer, or single-cell TCR sequencing studies. The autoantibody literature already cited in this review provides indirect evidence that humoral responses against tumor-derived antigens can arise in GBC patients, but those signatures are dominated by overexpressed self-proteins and tumor-associated antigens rather than by formally validated mutation-derived neoepitopes.

Translational evidence for pMHC-directed therapeutics in GBC is, at the time of this review, essentially absent. We found no published clinical or preclinical reports of TCR-mimic antibodies, TCR-mimic ADCs, or pMHC-directed bispecific constructs evaluated specifically in gallbladder cancer. The most mature regulatory experience with pMHC-directed redirection—tebentafusp, an HLA-A*02:01–restricted ImmTAC TCR-based bispecific approved in metastatic uveal melanoma—derives from a TCR-based, not antibody-based, platform and from a tumor histology unrelated to GBC [42]. Antibody-based programs targeting intracellular MHC-presented epitopes have been reported in other solid tumors and encompass the following two conceptually distinct categories: true mutation-derived neoantigens, exemplified by a TCR-mimic antibody against the TP53 R175H peptide presented on HLA-A*02:01, validated only in preclinical proof-of-concept work [43]; and overexpressed wild-type intracellular tumor-associated antigens, exemplified by the WT1 RMF/HLA-A*02:01 epitope addressed by a bispecific T-cell engager in early translational studies [16]. To our knowledge, neither category has been evaluated in gallbladder or wider biliary tract cancer cohorts.

Taken together, there is a coherent biological rationale to interrogate neoantigens and pMHC-presented peptides as therapeutic vulnerabilities in GBC, particularly in tumors with elevated mutational burden or MSI-H phenotypes and in patients harboring shared driver mutations such as *TP53* alterations that may, in principle, generate predictable HLA-restricted neoepitopes [6,27,41]. Surface-expressed targets such as HER2/*ERBB2* belong to a conceptually different category—that of canonical TAAs accessible to conventional antibodies, bispecific antibodies, and ADCs—and should not be invoked as part of the neoantigen/pMHC argument, even when they coexist in the same tumor. Specific evidence supporting TCR-mimic bispecifics or TCR-mimic ADCs in GBC is currently absent from the published literature, and this field should be framed as a hypothesis-generating research priority rather than as an emerging clinical strategy.

## 5. Standard of Care for Advanced or Metastatic Biliary Tract Cancer

Unresectable or metastatic BTC has a poor prognosis, with a median survival of approximately one year [4]. The most active cytotoxic chemotherapy agents for advanced BTC are cisplatin–gemcitabine-based regimens [4,44]. The current first-line standard of care was established by the ABC-02 trial (410 patients; 149 with GBC), in which cisplatin–gemcitabine (cisplatin 25 mg/m^2^ followed by gemcitabine 1000 mg/m^2^ IV, on days one and eight of 21-day cycles, for up to eight cycles) demonstrated a significant improvement in overall survival (OS) and progression-free survival (PFS) compared with single-agent gemcitabine (1000 mg/m^2^ IV, on days one, eight, and 15 of 28-day cycles, for up to six cycles) in patients with advanced or metastatic BTC (median OS 11.7 vs. 8.1 months; HR 0.64; 95% CI 0.52–0.80; *p* < 0.001; median PFS 8.0 vs. 5.0 months; *p* < 0.001). Treatment was administered for up to 24 weeks or until progression, unacceptable toxicity, or patient/clinician decision (Table 1) [4]. The Japanese randomized phase II BT22 trial [10] (84 randomized patients, 83 treated; 32 with GBC; recruited September 2006 to October 2008 across nine Japanese centers) showed results similar to ABC-02 for the cisplatin–gemcitabine combination (cisplatin 25 mg/m^2^ followed by gemcitabine 1000 mg/m^2^ IV, on days one and eight of 21-day cycles; median six cycles administered, up to 16 cycles/48 weeks maximum) versus gemcitabine monotherapy (1000 mg/m^2^ IV, on days one, eight, and 15 of 28-day cycles; median four cycles administered, up to 12 cycles/48 weeks maximum), administered until disease progression, intolerable toxicity, or withdrawal: median OS 11.2 vs. 7.7 months; median PFS 5.8 vs. 3.7 months; HR for OS 0.69 (95% CI: 0.42–1.13); and ORR 19.5% vs. 11.9% (Table 1) [10]. However, in the ABC-02 and BT22 studies, reported objective response rates (ORRs) were only 26% and 19.5% respectively [4,10]. Based on these results, Cis-Gem was for many years the established standard first-line treatment for patients with advanced BTC [4].

More recently, the addition of immune checkpoint inhibitors to the Cis-Gem doublet has prolonged median survival in patients with metastatic or locally advanced unresectable BTC [11,12]. The addition of the PD-L1 inhibitor durvalumab to the cisplatin–gemcitabine doublet in the phase III randomized, double-blind, placebo-controlled TOPAZ-1 trial (685 patients; ~25% with GBC) achieved improvements in PFS and OS. In this trial, which did not select participants by biomarker expression, patients receiving cisplatin 25 mg/m^2^ + gemcitabine 1000 mg/m^2^ IV, on days one and eight of 21-day cycles (for up to eight cycles), plus durvalumab 1500 mg IV every 3 weeks during chemotherapy, followed by durvalumab 1500 mg IV every 4 weeks as maintenance until progression or unacceptable toxicity, achieved a median PFS (mPFS) of 7.2 months and median OS (mOS) of 12.8 months, compared with mPFS of 5.7 months and mOS of 11.5 months, respectively, for participants receiving Cis-Gem plus placebo (HR for OS 0.80; 95% CI 0.66–0.97; and *p* = 0.021). Median exposure to study treatment was 7.3 months for durvalumab and 5.1 months for chemotherapy (Table 1) [11]. Based on these results, Cis-Gem plus durvalumab became a standard of care for patients with metastatic or locally advanced unresectable BTC who had not received systemic therapy for their advanced disease [11]. The randomized phase III KEYNOTE-966 trial (1069 patients; stratified by region, stage, and site of origin, gallbladder vs. intrahepatic vs. extrahepatic) demonstrated that the addition of the PD-1 inhibitor pembrolizumab to the cisplatin–gemcitabine doublet provides a similar survival benefit to that observed with durvalumab. Patients received pembrolizumab 200 mg IV every 3 weeks (for up to 35 cycles) plus gemcitabine 1000 mg/m^2^ IV on days one and eight every 21 days (until disease progression) and cisplatin 25 mg/m^2^ IV on days one and eight every 21 days (for <8 cycles), compared with placebo plus the same chemotherapy regimen. Median OS was 12.7 months in the pembrolizumab arm vs. 10.9 months in the placebo arm (HR 0.83; 95% CI: 0.72–0.95; and one-sided *p* = 0.0034), with median PFS of 6.5 vs. 5.6 months, confirming that the addition of immune checkpoint inhibitors to standard chemotherapy can be effective in this population (Table 1) [12]. Even with this advance in care, mOS remains at approximately one year, and 5-year OS data from the TOPAZ-1 and KEYNOTE-966 trials are not yet available [11,12].

## 6. Monoclonal Antibodies and ADCs: Clinical Evidence and Challenges

The translation of tumor biology into oncological practice is evidenced in the evaluation of targeted therapies, although most results emanate from global BTC cohorts in which GBC is only a subgroup [1].

Before reviewing the clinical evidence in GBC, it is useful to outline the structural and mechanistic categories of the antibody-based agents discussed in the following subsections. Bispecific antibodies are engineered immunoglobulin-derived molecules that incorporate two distinct antigen-recognition domains within a single construct, enabling simultaneous engagement of two epitopes either on the same target (biparatopic binding) or on two different molecules or cells [45].

**Figure 2:**
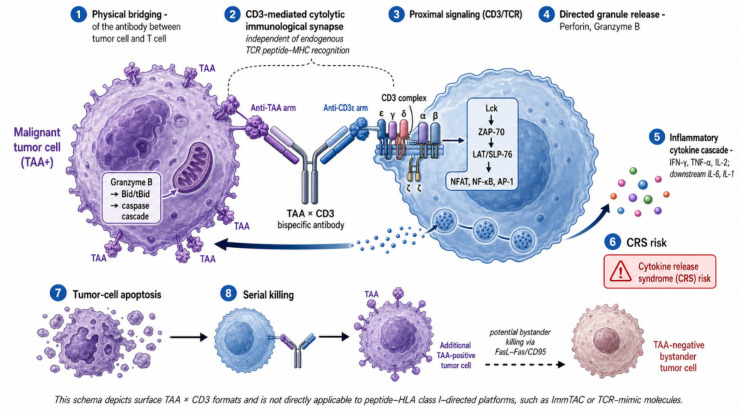
**Mechanism of action of CD3-engaging T-cell-redirecting bispecific antibodies.** Bispecific T-cell engagers combine a tumor-associated antigen (TAA)-binding arm directed against a malignant-cell surface antigen with an anti-CD3ε arm that recruits cytotoxic T cells, promoting (1) physical bridging between tumor cells and T cells; (2) formation of a CD3-mediated cytolytic immunological synapse that is independent of antigen-specific peptide–MHC recognition by the endogenous TCR, although downstream signaling is transmitted through the CD3/TCR complex; (3) proximal T-cell signaling through Lck, ZAP-70 and LAT/SLP-76, leading to activation of NFAT, NF-κB and AP-1; (4) polarized release of cytotoxic granules containing perforin and granzyme B, with granzyme B-mediated caspase activation and cleavage of Bid to tBid, thereby engaging the mitochondrial apoptotic pathway; (5) induction of an inflammatory cytokine cascade, including T-cell-derived cytokines such as IFN-γ and TNF-α and downstream myeloid-associated mediators such as IL-6 and IL-1; (6) potential risk of cytokine release syndrome; (7) tumor-cell apoptosis; and (8) serial killing of additional antigen-positive tumor cells after T-cell disengagement and re-engagement. In selected contexts, activated T cells may also induce limited bystander killing of nearby antigen-negative cells, potentially through FasL–Fas/CD95 signaling, although this is not the dominant canonical mechanism of TAA × CD3 activity. This schema specifically depicts surface antigen-directed TAA × CD3 formats and is not directly applicable to peptide–HLA class I-directed platforms, including TCR-based ImmTAC molecules such as tebentafusp or TCR-mimic antibodies, whose architecture and HLA restriction differ conceptually.

Conceptually, two functional families are particularly relevant. The first comprises dual-receptor or dual-pathway blockers; in the BTC field this category is exemplified by zanidatamab, a true bispecific antibody that biparatopically engages two extracellular domains of HER2, and—at the level of function rather than structure—by bintrafusp alfa, which is not a bispecific antibody in the strict structural sense but a bifunctional fusion protein consisting of an anti–PD-L1 IgG1 monoclonal antibody fused at its C-terminus to the extracellular domain of TGF-βRII acting as a TGF-β trap. The second family comprises T-cell engagers, in which one arm binds a tumor-associated antigen (TAA) on the malignant cell and the other arm recruits cytotoxic T cells, most commonly through CD3, to drive a CD3-mediated cytolytic synapse independent of native peptide–MHC recognition by the endogenous TCR [45,46]. Cell-surface antigens are particularly amenable to recognition by conventional and bispecific antibodies because their extracellular domains are sterically accessible and may support receptor clustering, effector engagement, or internalization depending on the target and antibody format. In solid tumors, however, the practical performance of these constructs is constrained by antigen heterogeneity, variable target density, on-target/off-tumor toxicity, immune exclusion in stroma-rich phenotypes, and the broader immunosuppressive features of the tumor microenvironment [34,36,46] (Figure 2).

**Figure 3:**
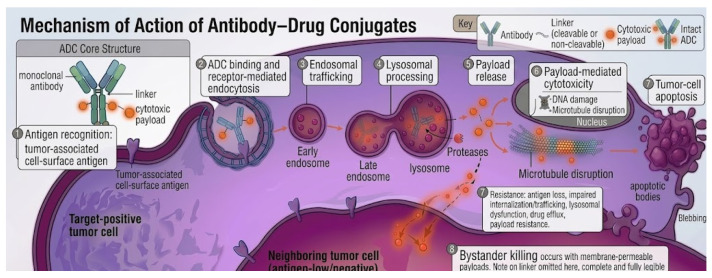
**Mechanism of action of antibody–drug conjugates (ADCs).** Core ADC structure comprises a monoclonal antibody, a linker (cleavable or non-cleavable) and a cytotoxic payload. Sequential steps depict (1) recognition of the tumor-associated cell-surface antigen, (2) receptor-mediated endocytosis, (3) endosomal trafficking, (4) lysosomal processing with enzymatic or pH-dependent payload release, (5) intracellular payload release, (6) payload-mediated cytotoxicity through DNA damage or microtubule disruption, and (7) tumor-cell apoptosis. Cleavable linkers liberate a membrane-permeable payload that may also kill neighboring antigen-low or antigen-negative tumor cells (bystander killing). Reported resistance mechanisms include downregulation or loss of surface antigen expression, impaired internalization or trafficking, lysosomal dysfunction, upregulation of drug-efflux transporters and intrinsic resistance to the payload class.

Antibody–drug conjugates (ADCs) couple a target-binding immunoglobulin to a cytotoxic payload through a chemical linker. Their canonical mechanism involves antigen recognition at the cell surface, receptor-mediated endocytosis, endosomal/lysosomal trafficking, intracellular release of the payload, and subsequent cytotoxicity, typically through DNA damage or microtubule disruption [9,47]. Linker chemistry is a key determinant of pharmacological behavior as follows: cleavable linkers (enzymatically or pH-sensitive) liberate a membrane-permeable payload that can also kill neighboring antigen-low cells—the so-called bystander effect, which has been invoked to rationalize the activity of trastuzumab deruxtecan in heterogeneously HER2-expressing tumors—whereas non-cleavable linkers require complete lysosomal degradation of the antibody and yield a charged catabolite with minimal bystander activity [47]. Reported mechanisms of resistance include downregulation or loss of surface antigen expression, impaired internalization or trafficking, lysosomal dysfunction, upregulation of drug-efflux transporters, and intrinsic resistance to the payload class [9,47] (Figure 3).

A conceptually distinct strategy targets peptide–MHC class I (pMHC-I) complexes rather than native cell-surface proteins. While conventional antibodies are largely confined to extracellular epitopes, TCR-mimic (also termed TCR-like) antibodies recognize short peptides—including those derived from intracellular or otherwise “undruggable” oncoproteins—when displayed on HLA class I molecules, thereby expanding the target universe to the same antigenic landscape surveyed by CD8^+^ T cells [16,40]. Conceptually related, although structurally distinct, are TCR-based bispecific platforms in which a soluble affinity-enhanced T-cell receptor—rather than an antibody—is fused to an anti-CD3 scFv to redirect T cells against pMHC complexes; tebentafusp, an HLA-A*02:01-restricted gp100-pMHC × CD3 ImmTAC molecule approved in metastatic uveal melanoma, is the regulatory prototype of this TCR-based class and should not be conflated with conventional bispecific antibodies [42]. Antibody-based pMHC-directed formats currently under exploration include TCR-mimic bispecific T-cell engagers and, at a more exploratory stage, pMHC-directed TCR-mimic antibody–drug conjugate strategies and chimeric antigen receptor constructs, most of which remain in preclinical or early translational development with limited or no published clinical activity to date [16,40]. The following translational limitations of all these platforms are non-trivial: HLA restriction confines eligibility to defined allelic subgroups, the surface density of any given pMHC complex is typically several orders of magnitude lower than that of canonical TAAs, off-target recognition of structurally related self-peptides has caused severe toxicity in early trials, and the development of high-affinity yet specific binders remains demanding [40,42].

### 6.1. The Anti-EGFR Rationale and Its Clinical Limitations

A proportion of patients with GBC harbor tumors with wild-type KRAS. Historically, based on extrapolations from colorectal cancer, it was hypothesized that this would confer sensitivity to anti-EGFR monoclonal antibodies (cetuximab or panitumumab). However, clinical translation in BTC proved disappointing [48]. Meta-analyses of randomized controlled trials concluded that the addition of anti-EGFR antibodies to first-line chemotherapy did not significantly improve PFS (HR = 0.88; 95% CI: 0.73–1.08) nor OS (HR 0.82; 95% CI 0.64–1.06) and was associated with an increase in serious hematological and cutaneous toxicities. Therefore, this therapeutic pathway lacks a standard role in current GBC treatment [48].

### 6.2. HER2-Targeted Therapies: Bispecific Antibodies and ADCs

HER2 assessment in BTC, including GBC, remains methodologically heterogeneous across studies, with positivity variably defined by immunohistochemistry, in situ hybridization, or next-generation sequencing. This variability is clinically relevant when interpreting response rates across HER2-directed trials and regulatory indications [7].

HER2 receptor alteration is one of the most consistent therapeutic targets. Given the suboptimal results with simple antibodies, the field has advanced toward more complex drug designs, including bispecific antibodies and ADCs [9,49].

Zanidatamab is a bispecific antibody with biparatopic geometry that recognizes two non-overlapping epitopes of the HER2 extracellular domain—the dimerization domain (ECD2, the binding site of pertuzumab) and the juxtamembrane domain (ECD4, the binding site of trastuzumab)—and binds in trans as follows: a single molecule bridges two adjacent HER2 receptors on the cell surface, providing the structural basis for receptor clustering, internalization, downregulation, and enhanced antibody-dependent cellular cytotoxicity (ADCC). This trans-binding mode, absent in the trastuzumab + pertuzumab combination, where each antibody engages a distinct HER2 receptor in cis, underlies the preclinical antitumor superiority of zanidatamab over both trastuzumab monotherapy and the trastuzumab–pertuzumab combination. The structural dependence on spatially proximal HER2 receptors provides a plausible biological rationale for the greater clinical activity observed in HER2 IHC 3+ tumors compared with HER2 IHC 2+ tumors [49].

Evidence from cohort 1 of the phase IIb HERIZON-BTC-01 trial (87 enrolled patients; 80 in HER2-positive cohort 1 (defined as ERBB2 amplification confirmed by central ISH with HER2 IHC 2+ or 3+), 41 [51%] of whom had GBC; and recruited at 32 centers in nine countries. Patients received zanidatamab 20 mg/kg intravenously on days one and 15 of each 28-day cycle, until disease progression, unacceptable toxicity, consent withdrawal, or physician decision (median six cycles administered; IQR 2–9; range 1–21; and median treatment duration of 5.1 months, IQR 1.9–8.8) (Table 1) [50].

In HERIZON-BTC-01, eligibility mandatorily required HER2/ERBB2 amplification confirmed by central ISH (HER2/Chr17 ratio ≥ 2.0, VENTANA HER2 Dual ISH DNA Probe Cocktail assay), and IHC (investigational VENTANA HER2/neu 4B5 assay) was used solely to assign patients to the following prospectively defined cohorts: cohort 1 (HER2 IHC 2+ or 3+; HER2-positive) and cohort 2 (HER2 IHC 0 or 1+; HER2-negative and HER2-low, respectively). Clinical activity was markedly dependent on protein expression as follows: the confirmed objective response rate by independent central review in cohort 1 was 51.6% (95% CI 38.6–64.5; 32/62) in HER2 IHC 3+ tumors versus 5.6% (95% CI 0.1–27.3; 1/18) in IHC 2+ tumors, with an overall cohort-1 ORR of 41.3% (95% CI 30.4–52.8), a median duration of response of 12.9 months and median PFS of 5.5 months [50]. This expression-dependent activity supported the FDA’s decision to restrict the accelerated approval of zanidatamab-hrii to adult patients with previously treated unresectable or metastatic HER2-positive biliary tract cancer confirmed as IHC HER2 3+, in whom longer follow-up showed an ORR of approximately 52% and a median duration of response of 14.9 months. This restriction generates a clinically relevant mismatch with real-world cohorts based on NGS, where positivity is defined by ERBB2 amplification and where concordance between IHC HER2 3+ and NGS-based amplification is imperfect in BTC, particularly in GBC, in which variable proportions of NGS-amplified ERBB2 tumors that do not reach IHC HER2 3+ have been described and therefore do not formally qualify for the approved zanidatamab indication. This decoupling between molecular and immunohistochemical criteria underscores the need to prospectively harmonize diagnostic algorithms in BTC and to adequately report the HER2 selection methodology used in each study [51].

Trastuzumab deruxtecan (T-DXd): an antibody–drug conjugate directed against HER2 with a cytotoxic payload (deruxtecan, a topoisomerase I inhibitor) connected by a cleavable linker [52]. Its activity in HER2-expressing BTC has been investigated in the biliary tract cancer cohort of the open-label, multicenter, multicohort phase II DESTINY-PanTumor02 trial, final analysis reported at ESMO 2025 including 267 patients in Part 1; and BTC cohort with 41 patients HER2-expressing IHC 3+/2+ BTC, previously treated. Patients received T-DXd 5.4 mg/kg IV every 3 weeks, until disease progression, unacceptable toxicity, or physician decision; in the overall study population (N = 267), the median number of cycles administered was 8.0 cycles, with a median total treatment duration of 5.6 months (range 0.4–41.9), and 21.7% of patients receiving ≥18 cycles (≈12 months of treatment) (Table 1) [53].

In the BTC cohort, the investigator-assessed confirmed objective response rate was 22.0% overall, with marked dependence on protein expression level as follows: 56.3% in HER2 IHC 3+ tumors by central testing (9/16) versus 18.8% in IHC 2+ tumors. Median PFS in the BTC cohort was 4.6 months (95% CI 3.1–6.0), reaching 7.4 months in the central IHC 3+ subgroup, and median OS was 7.0 months (95% CI 4.6–10.2), reaching 12.4 months in the central IHC 3+ subgroup. On the basis of pooled evidence from DESTINY-PanTumor02, DESTINY-Lung01, and DESTINY-CRC02 (192 adult patients with previously treated HER2-positive IHC 3+ solid tumors), T-DXd received tumor-agnostic FDA accelerated approval on 5 April 2024, for adult patients with previously treated unresectable or metastatic HER2-positive (IHC 3+) solid tumors with no satisfactory alternative treatment options, an indication that formally includes HER2-positive IHC 3+ biliary tract cancer. This makes T-DXd the first anti-HER2 therapy available for BTC under a tumor-agnostic framework, complementing the BTC-specific zanidatamab indication [53].

Although these approvals are clinically valuable, it must be noted that in both cases they derive from cohorts in which GBC was analyzed jointly with other biliary tract tumors, and comparative phase III long-term survival data are still pending [17,50,51,53].

### 6.3. Antiangiogenic Modulation: Considerations on Bevacizumab

The use of the anti-VEGF-A monoclonal antibody bevacizumab has been studied under the preclinical hypothesis of “vascular normalization.” It is postulated that transient VEGF inhibition could reorganize tumor capillaries, theoretically reducing interstitial pressure and facilitating chemotherapy penetration [54].

At the clinical level, the applicability of this concept in BTC is equivocal. Some retrospective analyses suggested favorable responses when bevacizumab was added to the cisplatin–gemcitabine regimen. In a real-world retrospective observational study [55] including 30 patients with advanced BTC, recruited between August 2018 and March 2021) evaluated the modified A-GC regimen, in which patients received bevacizumab 10 mg/kg IV every 21 days (administered 1 day before chemotherapy during the first two cycles as preconditioning, and concomitantly from cycle three onwards), combined with gemcitabine 1000 mg/m^2^ + cisplatin 25 mg/m^2^ IV on days one and eight of 21-day cycles, until disease progression, intolerable toxicity, or patient decision (median six cycles administered). This regimen achieved an objective response rate of 50.0% and a disease control rate of 80.0%, with median PFS of 8.4 months and median OS of 13.6 months. The following two critical limitations of this evidence must be emphasized: first, the retrospective single-center design with no control group; and second, the cohort comprised almost exclusively patients with intrahepatic cholangiocarcinoma (28/30; 93.3%) and did not include patients with gallbladder cancer, limiting direct extrapolability of these results to the GBC population [55]. However, the randomized phase II, double-blind, global, proof-of-concept IMbrave 151 trial (including 162 randomized patients: 79 to atezo+bev+CisGem and 83 to atezo+placebo+CisGem; and stratified by anatomical site [iCCA vs. eCCA vs. GBC], metastatic stage, and geographic region) was the first study designed to evaluate dual PD-L1/VEGF blockade on a chemotherapy backbone in untreated advanced BTC. Patients received, during the induction phase (up to eight cycles of 21 days), atezolizumab 1200 mg IV on day one + bevacizumab 15 mg/kg IV on day one + cisplatin 25 mg/m^2^ IV on days one and eight + gemcitabine 1000 mg/m^2^ IV on days one and eight (Arm A), compared with the same regimen substituting bevacizumab for placebo (Arm B); during the continuation phase (from cycle nine onwards), patients received atezolizumab 1200 mg + bevacizumab/placebo every 21 days, until disease progression or unacceptable toxicity. In the updated final analysis, median PFS was 8.35 months for atezo/bev vs. 7.9 months for atezo/plb (HR 0.67; 95% CI 0.46–0.95), failing to demonstrate an OS benefit (median OS 14.9 vs. 14.6 months; HR 0.97; and 95% CI 0.64–1.47), with confirmed ORR of 26.6% vs. 26.5% and median DOR of 10.28 vs. 6.18 months. The authors explicitly acknowledge that this signal-seeking trial is limited by small sample size and non-comparative design, making the findings hypothesis-generating rather than confirmatory (Table 1) [18].

## 7. Bispecific Immunotherapy in Advanced BTC

The activity of anti-PD-1 monotherapy in refractory advanced BTC was evaluated in the pooled analysis of KEYNOTE-158 (phase II tumor-agnostic; n = 104, all-comers for PD-L1) and KEYNOTE-028 (phase Ib; n = 24, all PD-L1-positive) [13]. Patients received pembrolizumab 200 mg IV every 3 weeks (KN-158) or 10 mg/kg IV every 2 weeks (KN-028), for a maximum of 2 years or until progression/toxicity. The BICR-confirmed ORR was 5.8% (6/104; 95% CI 2.1–12.1) in KN-158 and 13.0% (3/23) in KN-028, with median OS of 7.4 and 5.7 months respectively, and grade 3–5 TRAEs in 13.5% and 16.7%. The following three critical limitations qualify these results: absence of comparator, lack of specific tumor-site information (gallbladder, ICC, or ECC) explicitly acknowledged by the authors, and differing PD-L1 IHC assays between studies; together, they support that anti-PD-1 monotherapy has limited activity and should not be the preferred treatment for BTC outside clinical trials, justifying the subsequent development of chemo-immunotherapy combinations (TOPAZ-1, KEYNOTE-966) (Table 1) [13]. Dual CTLA-4 + PD-1 blockade was evaluated specifically in refractory advanced gallbladder cancer in the prospective, multicenter, open-label phase II SWOG S1609 DART cohort 48 trial [14] (including 19 GBC patients, 79% female, and median two prior lines), in which patients received nivolumab 240 mg IV every 2 weeks + ipilimumab 1 mg/kg IV every 6 weeks until disease progression or unacceptable toxicity. The confirmed BICR-assessed ORR was 16% (3/19; 1 CR + 2 PR), with response durations of 35+, 16, and 13 months, whereas the unconfirmed ORR and clinical benefit rate were both 32%; median OS was 7.0 months (95% CI 3.9–19.1) and 6-month PFS, 26%. Toxicity was manageable, with AST elevation as the most frequent grade 3/4 event (11%), although one death from hepatic failure possibly related to treatment was reported. Despite a modest increase in antitumor activity over anti-PD-1 monotherapy, the inherent limitations of trial design (small n, no comparator) and grade 3–4 toxicity motivate the evaluation of next-generation bispecific molecules co-targeting PD-1 and CTLA-4 with optimized pharmacokinetic profiles (e.g., AK104/cadonilimab, MEDI5752/volrustomig) [14]. To attempt to improve these margins, next-generation bispecific molecules are being evaluated.

### 7.1. Bintrafusp Alfa

Bintrafusp alfa is an investigational bifunctional fusion protein that integrates an anti-PD-L1 domain with an extracellular trap for the TGF-β receptor RII [56]; its theoretical objective is to focally neutralize the profibrotic pathways dependent on TGF-β in the tumor microenvironment. This mechanistic rationale was clinically evaluated in the open-label, multicenter, single-arm phase II trial INTR@PID BTC 047 [15] conducted in 36 centers in 10 countries; including 159 patients with locally advanced or metastatic BTC who had failed or were intolerant to first-line platinum-based chemotherapy, including 32 with gallbladder cancer [20.1%], 95 with iCCA, and 32 with eCCA). Patients received bintrafusp alfa 1200 mg IV every 2 weeks until confirmed disease progression, unacceptable toxicity, or withdrawal, with a median treatment duration of 8.0 weeks (range 2.0–80.1; approximately four Q2W cycles) and median follow-up of 16.1 months. The IRC-confirmed ORR was 10.7% (95% CI 6.4–16.6), with three complete responses and 14 partial responses, median DOR of 10.0 months, median PFS of 1.8 months, and median OS of 7.6 months (6/12/18-month OS: 57.9%/38.8%/26.9%); responses were independent of PD-L1 expression. Notably, activity by BTC subtype showed ORR of 12.6% in iCCA, 9.4% in eCCA, and only 6.3% (2/32) in gallbladder cancer, limiting direct extrapolation to the GBC population. Grade ≥ 3 TRAEs occurred in 26.4% of patients, with one treatment-related death from hepatic failure. The study did not meet its primary endpoint, as the lower bound of the 95% CI (6.4%) was below the prespecified 10% threshold, leading to discontinuation of the monotherapy program and subsequent termination of the first-line phase II/III study due to lack of PFS improvement at interim analysis. Taken together, these results show that the clinical translation of the dual PD-L1/TGF-β blockade concept as monotherapy did not replicate the expected biological benefit in refractory BTC, particularly in GBC, motivating the development of next-generation bispecifics with optimized pharmacokinetic and mechanistic profiles [15].

### 7.2. Rilvegostomig and the TIGIT/PD-1 Pathway

Rilvegostomig is a bispecific antibody designed to jointly inhibit PD-1 and the co-inhibitory receptor TIGIT. Binding-characterization studies indicate that its bispecific structure optimizes checkpoint control by anchoring primarily to TIGIT, which increases PD-1 saturation through coordinated engagement and theoretically minimizes non-specific peripheral depletion [57]. In BTC, rilvegostomig is currently being evaluated in ARTEMIDE-Biliary02, a trial of rilvegostomig or durvalumab plus chemotherapy as first-line treatment for biliary tract cancer. At the editorial cut-off, the number of enrolled patients, the confirmed dose schedule, and the planned number of treatment cycles in the rilvegostomig arm have not been reported in peer-reviewed form. Given the immaturity of the evidence, any statement about its clinical impact must await the definitive results of phase III trials [58].

## 8. Perioperative Scenarios: Initial Exploration in Potentially Resectable Localized Disease

The high rate of postsurgical relapse in BTC has motivated the exploration of perioperative therapies. Although the field was historically dominated by retrospective series and early-phase studies, prospective randomized evidence is now emerging [19].

In the neoadjuvant setting, the objective is to induce tumor shrinkage to maximize R0 resections. The GAIN trial is a multicenter, randomized 1:1, open-label phase III study evaluating perioperative gemcitabine–cisplatin chemotherapy versus immediate radical surgery (with optional investigator’s-choice adjuvant therapy) in patients with incidental gallbladder carcinoma (pT2-3 N- or pT1-3 N+) after simple cholecystectomy and in resectable or borderline-resectable ICC/ECC. The experimental arm received gemcitabine 1000 mg/m^2^ plus cisplatin 25 mg/m^2^ i.v. on days one and eight every 3 weeks, for three preoperative and three postoperative cycles (six perioperative cycles total). The planned sample size was N = 333 (powered at 80%, one-sided α 0.05, to detect HR 0.70 over a 24-month control-arm median OS); the primary endpoint was overall survival, with PFS, R0-resection rate, QoL, toxicity (CTCAE v5.0) and 30-/90-day perioperative morbidity–mortality as secondary endpoints. However, the trial closed prematurely owing to slow accrual, with only 68 patients enrolled, so its findings must be regarded as strictly hypothesis-generating. At the editorial cut-off, definitive peer-reviewed results are not yet available, and any conclusion on the clinical impact of perioperative GemCis in IGBC and resectable BTC must await final publication (Table 1) [19].

The POLCAGB trial is a phase III randomized 1:1, open-label single-institution study (Tata Memorial Center, Mumbai) comparing neoadjuvant chemotherapy (NACT) versus neoadjuvant chemoradiation (NACRT) in patients with locally advanced gallbladder adenocarcinoma (LAGBC) initially unsuitable for R0 resection (T3/T4 with liver infiltration 2–5 cm, N1, type I/II biliary obstruction, duodenal/colonic abutment without mucosal infiltration, or <180° vascular involvement). The NACT arm received gemcitabine 1000 mg/m^2^ (days one, eight) plus cisplatin 25 mg/m^2^ (day one) every 3 weeks for four cycles; the NACRT arm received 52–57 Gy in 25 fractions with concurrent weekly gemcitabine 300 mg/m^2^, followed by two cycles of gemcitabine–cisplatin at the same NACT doses; both arms received three postoperative adjuvant cycles of gemcitabine–cisplatin at the same schedule. The planned sample size was 314 patients (HR 0.70, two-sided α 0.05, and β 0.20), with overall survival (OS) as primary endpoint and event-free survival (EFS), R0-resection rate and Clavien–Dindo postoperative morbidity as secondary endpoints. Owing to slow accrual, an ethics-approved interim analysis included 124 patients (64 NACT vs. 60 NACRT) enrolled between October 2016 and September 2024, with 93 OS events and 62-month median follow-up. In the intention-to-treat analysis, NACRT significantly improved median OS (21.8 vs. 10.1 months; HR 0.56, 95% CI 0.37–0.84; and *p* = 0.006), EFS (10.6 vs. 4.89 months; HR 0.58, 95% CI 0.39–0.85; and *p* = 0.006) and R0 rate (51.6% vs. 29.7%; *p* = 0.01), without significant excess of Clavien–Dindo ≥ 3 morbidity (28.1% vs. 18.2%; *p* = 0.30). As these data correspond to an interim analysis presented at ASCO 2025 and not yet to a peer-reviewed full publication, definitive conclusions should await final reporting (Table 1) [20,59].

Approaches integrating preoperative immunotherapy in GBC are of purely anecdotal or exploratory nature at this time [60].

In the adjuvant setting, capecitabine use is frequently supported by the results of the BILCAP trial [21], which is a multicenter, randomized 1:1, open-label phase III study in 44 UK centers comparing adjuvant capecitabine versus observation following macroscopically complete surgical resection of cholangiocarcinoma (intrahepatic, hilar or extrahepatic) or muscle-invasive gallbladder cancer in patients with ECOG ≤ 2. A total of 447 patients were randomized (223 capecitabine vs. 224 observation) between 2006 and 2014, with capecitabine 1250 mg/m^2^ orally twice daily on days 1–14 every 21 days for eight cycles (total 24 weeks of treatment). The primary endpoint was overall survival; secondary endpoints included recurrence-free survival, toxicity, quality of life and health economics. In the intention-to-treat analysis, median OS was 51.1 vs. 36.4 months (HR 0.81, 95% CI 0.63–1.04; *p* = 0.097), formally not meeting the primary endpoint; however, the prespecified sensitivity analysis adjusted for nodal status, grade and sex showed a significant benefit (HR 0.71, 95% CI 0.55–0.92; *p* < 0.01), and the per-protocol analysis confirmed median OS 53 vs. 36 months (HR 0.75, 95% CI 0.58–0.97; *p* = 0.028). Long-term follow-up at 106 months maintained the benefit (HR adjusted 0.74, 95% CI 0.59–0.94), and grade 3–4 toxicity was lower than anticipated. Despite the formally negative primary endpoint, the consistent benefit across sensitivity, per-protocol and long-term analyses led to capecitabine being adopted as the international adjuvant standard of care for resected biliary tract cancer (Table 1) [21]. To attempt to reduce recurrence, large consortia are evaluating checkpoint blockades. The global prospective phase III ARTEMIDE-Biliary01 trial is recruiting an estimated n = 750 patients with resected BTC (R0/R1, ECOG 0-1) to compare investigator’s choice—standard adjuvant chemotherapy (capecitabine, S-1, or gemcitabine–cisplatin for up to 6 months) combined with rilvegostomig (anti-PD-1/anti-TIGIT bispecific antibody, IV every 3 weeks for up to 1 year) versus the same chemotherapy plus placebo, with recurrence-free survival as the primary endpoint and overall survival as the key secondary endpoint. The primary completion of this trial (estimated for 2029) will be imperative to determine whether modulating antibodies have proven clinical utility in prophylaxis (Table 1) [61].

## 9. Limitations and Challenges in GBC Research

Despite the progress described, the GBC literature faces severe methodological limitations [2] as follows:

- Extrapolation Bias in BTC: In clinical trials (“basket trials”), GBC usually represents a small fraction of the cohort analyzed [50,62]. Assuming that the therapeutic responses observed in global BTC apply equally to GBC ignores its profound anatomopathological and mutational differences [1,6,7,63].
- Data Immaturity: Strategies such as autoantibody signatures [32,33], TIGIT inhibition [57,58], and perioperative protocols rest on proofs of concept [19,59], requiring mandatory validation before any clinical adoption.
- Geographic Inequality: GBC being an orphan pathology in North America and Europe [1,2], but endemic in Andean South American and Asian regions [1,2,23], there is a chronic deficit in recruitment equity for global trials [24,29], hindering validation of the impact of genetic ancestry on molecular heterogeneity and biomarker distribution [7,27].

## 10. Conclusions

Gallbladder cancer occupies the following paradoxical position in modern oncology: a tumor classified as “rare” in regulatory terms yet endemic in the Andean and South Asian corridors, where it remains one of the leading causes of cancer-related death in women under sixty. The biology that has emerged over the last decade—marked genomic heterogeneity, a stroma-rich and immunosuppressive microenvironment, recurrent *ERBB2* alterations of comparable prevalence across geographically distinct cohorts, and a generally low-to-intermediate tumor mutational burden—provides both the rationale and the constraints for the antibody-based therapeutic landscape reviewed here. This review has deliberately confined itself to antibody-based platforms; we acknowledge that the broader precision-oncology repertoire in BTC also includes FGFR2-fusion inhibitors, IDH1-mutant inhibitors, and MSI-H/dMMR-directed checkpoint blockade, all of which are predominantly relevant in cholangiocarcinoma and exceed the scope of this work. Within the antibody universe, no single axis of vulnerability has yet proved sufficient: HER2 stands out as the most consistent extracellular target, PD-1/PD-L1 inhibition delivers modest but reproducible survival gains, and neoantigen- or pMHC-directed strategies remain biologically attractive but clinically untested in this disease.

Translationally, the field has moved from cytotoxic intensification—which failed to improve survival in SWOG S1815—to immune-chemotherapy combinations such as TOPAZ-1 and KEYNOTE-966, and toward target-restricted antibody platforms exemplified by zanidatamab and trastuzumab deruxtecan, both of which received their first BTC-relevant regulatory indications between 2024 and 2025. The most clinically meaningful lesson of these approvals is that response is steeply expression-dependent: ORRs of ~52% in HER2 IHC 3+ versus ~6% in IHC 2+ tumors reframe HER2 in BTC as a quantitative rather than a binary biomarker. This gradient also exposes a regulatory mismatch with NGS-defined cohorts—where positivity is anchored in *ERBB2* amplification rather than in protein expression—and the discordance is particularly consequential for Latin American practice, in which targeted NGS panels are increasingly accessible while validated HER2 IHC remains unevenly available. In parallel, bispecific checkpoint modulators (rilvegostomig in ARTEMIDE-Biliary01 and -02), perioperative chemotherapy (GAIN, POLCAGB), and adjuvant capecitabine (BILCAP) collectively signal a transition from advanced-disease salvage toward earlier-stage immunological intervention—a shift whose ultimate value will be defined by phase III readouts expected between 2027 and 2029.

Looking forward, five research priorities stand out as decisive for the next phase of GBC drug development. First, prospective validation of multi-protein and autoantibody signatures—such as the Karolinska SomaScan-derived 13-protein LASSO panel—against histologically confirmed cholecystitis controls in endemic regions, where pretest probability is highest and where conventional CA 19-9/CEA underperform. Second, harmonization of HER2 assessment across IHC, ISH and NGS platforms, with explicit reporting of the diagnostic algorithm in every HER2-directed trial, to close the gap between regulatory eligibility and biological positivity. Third, dedicated GBC subgroup analyses, or ideally GBC-specific arms, in pivotal BTC trials, given that gallbladder tumors differ from intra- and extrahepatic cholangiocarcinomas in mutational repertoire, microenvironmental composition, and ancestral background and cannot be safely subsumed under a pooled BTC label. Fourth, rigorous interrogation of pMHC-directed and TCR-mimic platforms in GBC, framed as a hypothesis-generating priority rather than as a translated clinical strategy, given the absence of any published preclinical or clinical evidence in this histology to date. Fifth—and perhaps most distinctive of this disease—ancestry-aware characterization of the neoantigen repertoire and HLA landscape in Mapuche, Aymara, and other Amerindian-descended cohorts, where the prevailing in silico predictors of MHC-I peptide binding remain trained on databases skewed toward European ancestry, and where this bias risks systematically misclassifying the very neoepitopes most relevant to the populations carrying the highest disease burden.

Ultimately, the most enduring contribution of antibody-based therapeutics to gallbladder cancer will depend less on the next molecule and more on a structural commitment that the field has historically neglected: equitable inclusion of high-incidence populations in the trials that define our standards of care. The EULAT Eradicate GBC consortium, the Chilean and Bolivian cohorts feeding contemporary genomic atlases, and the South American sites participating in HERIZON-BTC-01 and ARTEMIDE-Biliary01 illustrate what this commitment looks like in practice. Until BTC trial enrollment matches BTC disease incidence, the antibody revolution we have described will continue to be designed in cohorts that look nothing like the patients who need it most—a gap that no molecule, regardless of how elegant its design, can close on its own.

## Figures and Tables

**Table 2:** Diagnostic and predictive biomarkers in GBC: operative performance and methodological characteristics.

Biomarker/Study	Clinical Purpose	Population/Comparator	Methodology	N	Sensitivity	Specificity	AUC	Key Limitation
**Part A: Diagnostic biomarkers**								
CA 19-9/CEA [31]	Metastatic triage	Newly diagnosed GBC	Serum measurement at presentation	1500	40.3%	89.1%	0.74	Low sensitivity precludes use as standalone screening tool
ANXA1 autoantibody [32]	Early detection	Early GBC vs. lithiasis controls	Serum autoantibody assay (single marker)	NR	41.7%	89.9%	0.69	Single autoantibody lacks operative performance for clinical screening
Soma Scan^®^ 7K plasma proteomics + ML [33]	Multi-protein panel	GBC vs. histologically confirmed AC	Aptamer platform (7596 proteins) + Elastic-Net + LASSO; 80/20 train-test + LOOCV	44 GBC vs. 38 AC	NR	NR	AUC 94–98%	No external validation; clinically relevant comparator (cholecystitis) but small sample; hypothesis-generating
**Part B: Predictive and tumor microenvironment biomarkers**								
PD-L1 in tumor cells [37]	Predictive—immunotherapy response	BTC including GBC	IHC SP142 clone; TC ≥ 2+ with ≥5% stained tumor cells	203	NR	NR	Positivity 12%	TC scoring (not CPS); reported variability across studies 9–72% depending on clone/threshold
PD-1 in TILs [37]	TME—adaptive immune activation	BTC including GBC	IHC NAT105 clone; threshold ≥ 1+	203	NR	NR	Positivity 55% in GBC	Suggests adaptive but exhausted immune microenvironment; predictive value undefined in absence of harmonized cut-offs

Abbreviations: AC: acute cholecystitis GBC = gallbladder cancer; BTC = biliary tract cancer; TILs = tumor-infiltrating lymphocytes; TME = tumor microenvironment; ML = machine learning; NR = not reported, TC = tumor proportion score.

## Data Availability

The original contributions presented in this study are included in the article. Further inquiries can be directed to the corresponding authors.
